# Paternal portrait of populations of the middle Magdalena River region (Tolima and Huila, Colombia): New insights on the peopling of Central America and northernmost South America

**DOI:** 10.1371/journal.pone.0207130

**Published:** 2018-11-15

**Authors:** Luz Angela Alonso Morales, Andrea Casas-Vargas, Madelyn Rojas Castro, Rafael Resque, Ândrea Kelly Ribeiro-dos-Santos, Sidney Santos, Leonor Gusmão, William Usaquén

**Affiliations:** 1 Populations Genetics and Identification Group, Institute of Genetics, Universidad Nacional de Colombia, Bogotá, Colombia; 2 Laboratório de Toxicologia e Química Farmacêutica, Departamento de Ciências da Saúde e Biológicas, Universidade Federal do Amapá, Macapá, Brazil; 3 Human and Medical Genetics Laboratory, Institute of Biological Sciences, Federal University of Pará (Universidade Federal do Pará - UFPA), Belém, state of Pará (PA), Brazil; 4 DNA Diagnostic Laboratory (LDD), Institute of Biology, State University of Rio de Janeiro (UERJ), Rio de Janeiro, Brazil; Xiamen University, UNITED STATES

## Abstract

The valley of the Magdalena River is one of the main population pathways in Colombia. The gene pool and spatial configuration of human groups in this territory have been outlined throughout three historical stages: the Native pre-Hispanic world, Spanish colonization, and XIX century migrations. This research was designed with the goal of characterizing the diversity and distribution pattern of Y-chromosome lineages that are currently present in the Tolima and Huila departments (middle Magdalena River region). Historic cartography was used to identify the main geographic sites where the paternal lineages belonging to this area have gathered. Twelve municipalities were chosen, and a survey that included genealogical information was administered. Samples collected from 83 male volunteers were analyzed for 48 Y-SNPs and 17 Y-STRs. The results showed a highly diverse region characterized by the presence of 16 sublineages within the major clades R, Q, J, G, T and E and revealed that 93% (n = 77) of haplotypes were different. Among these haplogroups, European-specific R1b-M269 lineages were the most representative (57.83%), with six different subhaplogroups and 43 unique haplotypes. Native American paternal ancestry was also detected based on the presence of the Q1a2-M3*(xM19, M194, M199) and Q1a2-M346*(xM3) lineages. Interestingly, all Q1a2-M346*(xM3) samples (n = 7, with five different haplotypes) carried allele six at the DYS391 locus. This allele has a worldwide frequency of 0.169% and was recently associated with a new Native subhaplogroup. An in-depth phylogenetic analysis of these samples suggests the Tolima and Huila region to be the principal area in all Central and South America where this particular Native lineage is found. This lineage has been present in the region for at least 1,809 (+/- 0,5345) years.

## Introduction

Similar to most South American populations, the genetic and cultural background of Colombians has been shaped by complex population dynamics: first, the peopling of Colombia by Native American inhabitants; second, the Spanish conquest and slave trade; and third, the different waves of migration and movements that characterized the XX century and the first decade of the XXI century [1,2]. Over time, Colombia has received genetic contributions from different Native American groups, European settlers and African slaves from different areas, resulting in high genetic diversity and a nonuniform composition of the current gene pool [3,4,5].

Several genetic studies have been performed in Colombia using different types of molecular markers (matrilineal, patrilineal, and autosomal) to analyze numerous populations in different regions of the country [3,5,6,7,8,9,10,11,12,13,14,15,16].

Such investigations have demonstrated that populations from urban areas usually present higher levels of non-Native admixture and uniparental-lineage diversity. In addition, these studies have shown how the proportions of Native American, African and European lineages vary throughout the country. The highest frequencies of African genetic contribution have been found on the Caribbean coasts and islands and in the Pacific Coast region. In contrast, Native lineages are found in higher proportions in Native populations, mainly from the eastern part of the country, including the Amazon and rural areas of southwest and northern Colombia. The European contribution is present throughout the country. This diversity reflects a nonuniform genetic pool in Colombian communities and regions.

In the history of Colombian peopling, three routes have been suggested: one following the Pacific coast, another traveling into the Colombian mountains (through the valleys of the Cauca and Magdalena Rivers) and the last, along the Atlantic coast [17].

**Fig 1:**
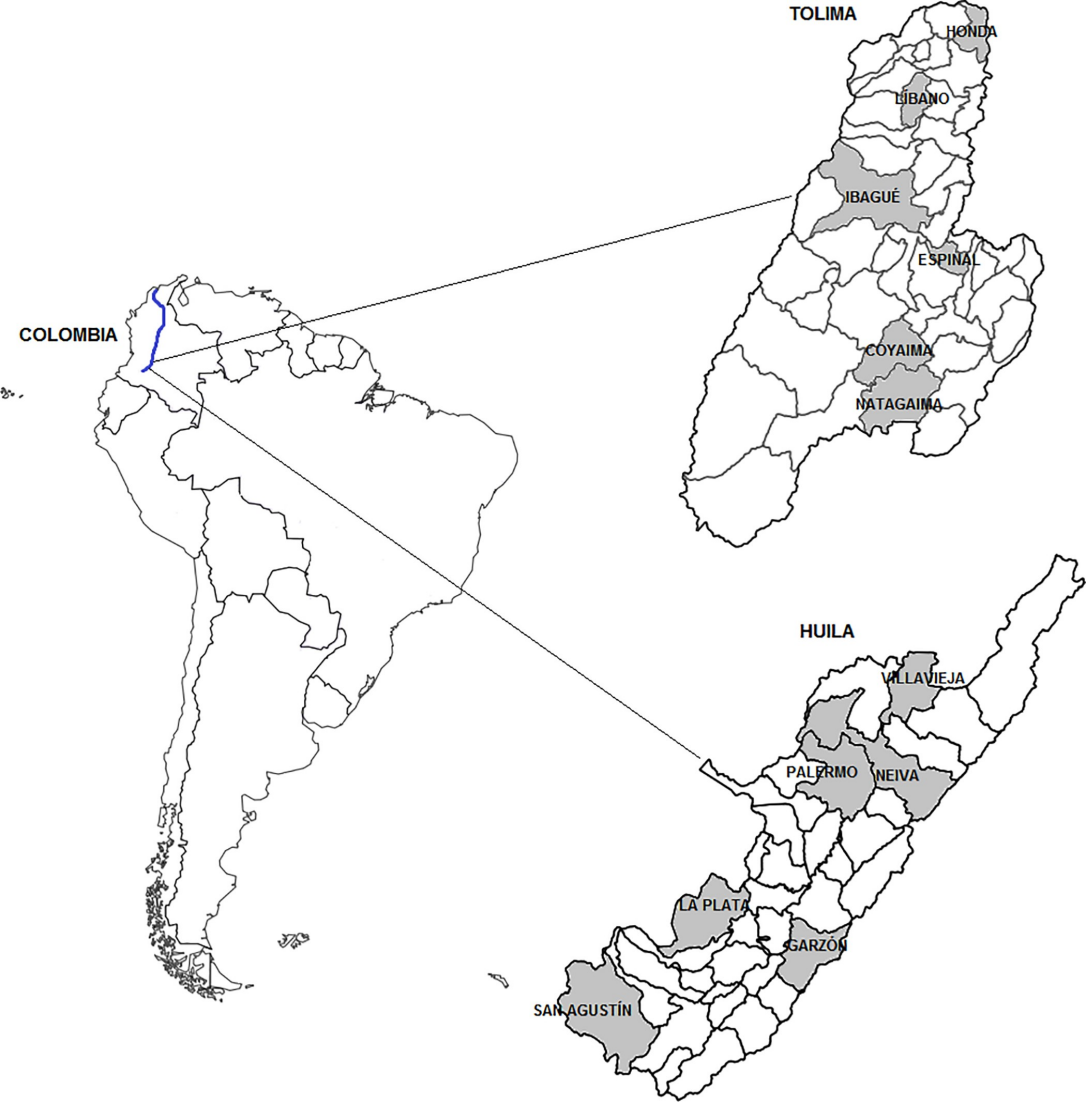
Geographic location of the Colombia and the Magdalena River in South America and the 12 sampling locations (in bold) in the departments of Tolima and Huila. Maps created with the QGis open source software [20], map data is available from https://geoportal.dane.gov.co/v2/?page=elementoDescargaMGN [21] and https://www.arcgis.com/home/item.html?id=d3d2bae5413845b193d038e4912d3da9 [22].

In this context, the valley of the Magdalena River is the main hydrographic system in Colombia and the major economic development backbone in the country [18]. Its basin covers a surface of 257.438 square kilometers (km^2^) and is densely populated. Nearly 79% of the entire population of Colombia lives in the Magdalena watershed, amounting to a demographic density of 120 inhabitants km^2^ [19]. The valley spans the final spurs of the Andes Mountain range at high altitudes to its delta in the Atlantic Ocean, in the north of the country, in low-lying areas (Fig 1).

This scenario of this region encompasses three key historical moments in the configuration of the Colombian territory: (i) the pre-Columbian Period, in which Native American groups used this region as a route of communication and source of food; (ii) the Colonial Period, in which this area served as a penetration route towards the center of the country; and (iii), the end of the XIX century, at which time the Magdalena River became the main fluvial route in the country and a transit path for the arrival of new settlers [1].

The area covered by the Tolima and Huila territory is part of the Magdalena River’s mid-valley. This region was inhabited by hunting, fishing and gathering groups 16,000 years ago [23]. The inventory of the existing archaeological sites in these departments represents the passage of time, beginning with the first hunter-gatherers who occupied this region, extending to the current farming and urban communities [24].

In pre-Columbian times, the region of Tolima and Huila was characterized by high diversity of Native groups, which were greatly reduced in size during the European colonization of America. The mineral resources of the region were the original reason for this colonization, but throughout the XVIII century, the exploitation of quinine brought a new migratory wave of settlers from Spain and from other regions of Colombia, followed by agricultural modernization [2].

According to the Regional Indigenous Corporation of Tolima (CRIT), from pre-Columbian times to the present day, two main Native communities have inhabited the Tolima and Huila territories. The “Pijao” ethnic group (known in Colombia for their resistance to Spanish domination) remains on their ancestral land, especially in the municipalities of Ortega, Coyaima, Natagaima, Chaparral, and Saldaña, with approximately 17,000 members. The “Nasa” group mainly resides in the Cauca and Huila departments, with some communities in Tolima. Currently, there are approximately 138,501 Nasa people in both departments [25].

The majority of genetic studies performed in Tolima and Huila have focused on evaluating diversity levels in the “Pijao” and “Nasa” Native communities using molecular markers such as autosomal STRs, restriction fragment length polymorphisms (RFLPs) in mitochondrial DNA (mtDNA) [26,27], Y-chromosome-specific SNPs [27,28,29,30], and autosomal SNPs [27,31]. In Huila, a single study was carried out in a sample of seven non-Native individuals from the municipality of Neiva, in which autosomal SNPs, X-chromosomal STRs, mtDNA and Y-chromosomal markers were investigated [3].

Since the last century, the Tolima and Huila territories have experienced waves of violence and forced displacement (incited by armed groups). This phenomenon has had a great impact on the demography of Native and rural communities in this area. Thus far, there has been no exhaustive evaluation of the diversity of the Y-chromosome lineages that are present in this territory (not only in individuals from Native communities) using a representative sample from both departments.

To characterize the patrilineages of the current inhabitants of the Tolima and Huila regions, this study focused on phylogenetic analysis of the Y-chromosome, using a synergic combination of markers defining haplogroups, such as single-nucleotide polymorphisms (SNPs) and microsatellites (short tandem repeats [STRs]), to evaluate migration, admixture, and ancestry patterns and to reveal additional microevolutionary processes associated with population structure.

## Materials and methods

### Study population

This study was conducted on samples collected by the Population Genetics and Identification Group of the Universidad Nacional de Colombia in the Tolima and Huila region in 2012.

Prior to the field phase, a historic cartography was performed on both departments to identify the main geographic sites where the paternal lineages that shaped the region have gathered over time, by the analysis of demographic, economic and political variables of the territory from the colony time to the present (all details are in S1 File). From this a priori investigation, 12 locations from both departments were selected as a reference sampling places (Honda, Líbano, Ibagué, Espinal, Coyaima, and Natagaima in Tolima; and Villavieja, Neiva, Palermo, Garzón, La Plata, and San Agustin in Huila).

A purposive sample was performed among these 12 locations, with a total sample size of 330 people. From it, 119 samples were from men. However, as a consequence of long periods of storage before this study was performed; 36 of the 119 samples showed poor DNA quality. For this reason, to ensure good data quality, the final sample size of this study is composed by 83 male samples from the most relevant historical settlement places were selected to analyze the paternal portrait of the Tolima and Huila populations.

Unfortunately, due to both departments have suffered from serious internal violence as a consequence of the presence of illegal defense forces, the population access is limited, for this reason, we could not increase the male sampling number.

Samples were collected at the selected location´s hospitals. Two inclusion criteria were used to select participants; the participants had to be at least 18 years old or older and had to have been a local area resident for at least one year. All samples were taken under written informed consent.

DNA extraction was performed from blood samples using the salting out method, following the DNA 2000 kit (CorpoGen) protocol.

This research was approved by the ethics committee of the Faculty of Science of the Universidad Nacional de Colombia.

### Genotyping of Y-chromosome STRs

Seventeen Y-chromosomal STR loci were amplified using the AmpFlSTR Yfiler PCR Amplification kit, following the manufacturer’s instructions (User’s Manual, Applied Biosystems). Separation and detection of the amplified products were performed in an Applied Biosystems PRISM 310 genetic analyzer. Allele assignations were performed by comparison with a reference ladder included in the kit, and using the nomenclature recommended by the International Society for Forensic Genetics (ISFG) [32]. These analyses were performed in the laboratory of the Population Genetics and Identification group, of the Genetics Institute of the Universidad Nacional de Colombia.

The Y- chromosomal data for all 83 donors have been submitted to the open-access Y-STR Haplotype Reference Database (YHRD, www.yhrd.org): accession number YA004510.

### Genotyping of Y-chromosome SNPs

To select the best set of SNP markers to be typed, Y-STR haplotype information was used to predict the most likely haplogroup of each sample, using an online tool (http://www.hprg.com/hapest5/). This software is based on a Bayesian approximation using allelic frequencies calculated from haplotype collections taken from published papers and databases [33]. This makes it possible to determine the probability of a Y-STR haplotype being found within a given haplogroup. The assigned haplogroup is the one to which a given haplotype has the highest probability of belonging.

The predicted haplogroups were then confirmed by genotyping 48 Y-chromosome SNPs, using the SNaPshot Kit (Applied Biosystems), in six previously described multiplex reactions including the markers: (i) SRY1532, M213, M9, M70, M22, Tat, 92R7, M173, and P25 [34]; (ii) M201, M170, 12f2, M26, M62, and M172 [34];(iii) M269, L23, U106, S116, U152, M529, M153, M167(SRY2627) [35];(iv) M12, M67, M92, M241, M410 [36], M267 [37]; (v) M3, M19, M194, M199, M242, M346 and P36.2 [12]; (vi) M96, M33, P2, M2, M154, M191, M35, M78, M81, M123, V6, M293, M85 [38].

For all multiplex reactions, PCRs were performed in a 10 μl volume containing 5 μl of the Qiagen multiplex PCR kit, 1 μl of primer mix (each primer at 2 μM), 3.5 μl of water, and 0.5 μl of DNA (5–20 ng/μl). The PCR thermocycling conditions consisted of an initial denaturation at 95°C for 15 minutes, followed by 35 cycles at 94°C for 30 seconds, 60°C for 90 seconds and 72°C for 60 seconds, and a final extension at 72°C for 10 minutes. After confirming the amplification in a polyacrylamide gel (T9%, C5), 1 μl of the PCR product was purified using 0.5μl of Exo-SAP-it (USB), and incubated at 37°C for 15 minutes, followed by inactivation of the enzyme at 85°C for 15 minutes.

The SNaPshot multiplex reactions were performed in 5 μl containing 2 μl of SNaPshot multiplex mix (Applied Biosystems), 1.5 μl of the purified PCR product and 1.5 μl of a mix of the single base extension (SBE) primers. The reaction protocol consisted on 25 cycles at 95°C for ten seconds, 50°C for five seconds and 60°C for 30 seconds. The final products were purified with 1μlof SAP (USB) at 37°C for one hour, followed by a denaturation step at 85°C for 15 minutes. The SBE products were run in an Applied Biosystems 3130 Genetic Analyzer and analyzed using the GeneMapper software v4.0, at the Human Genetics and Medicine Laboratory, Biological Sciences Institute at the UFPA (Federal -University of Pará), Brazil.

The haplogroup nomenclature was assigned according to van Oven et al. [39]

### Statistical analysis

An analysis of molecular variance (AMOVA) was performed using Arlequin software 3.5.1.2 [40] to evaluate the random distribution hypothesis for individuals among populations. Haplotype diversities were calculated according to Nei [41], using the following equation:

$$HD=\frac{n}{n-1}(1-\sum_{i=1}^{k}{p_{i}}^{2})$$ (1)

Where *n* is the sample size and *p_i_* is the frequency of the i-haplotype.

Phylogenetic relationships between Y-chromosome haplotypes among American and Asian populations were inferred for the Native American Q lineage (S1 Table) and visualized with the median-joining algorithm [42] using the Network software 4.6.1.1 (www.fluxustechnology.com), which assumes a stepwise mutation model for STRs. In this analysis, DYS385 was excluded because it corresponds to two loci that cannot be differentiated via the applied typing method [43], while for the DYS389II-I locus, the number of repeats in DYS389I was subtracted [44]. In the network, a weight of three to 10 was assigned to each locus, following the methodology proposed by Muzzio et al. [45] and using the mutation rates reported in the Y-haplotype reference database [46].

Estimates of the time to the most recent common ancestor (TMRCA) were determined using rho statistics, implemented in the NETWORK program, employing a mean effective mutation rate for Y-STRs of 6.9 × 10^−4^/locus/25 years [47]. The ancestral haplotype was inferred using the modal allele at each STR locus [48].

To compare the Y-chromosome haplogroup frequencies identified in this study with those from 30 other Colombian samples, a principal component analysis (PCA) was performed using Multiple Statistical Package (MVSP) software, version 3.1 [49].

## Results and discussion

### Y-chromosome diversity in the Tolima and Huila departments

The Y-chromosome haplotypes and haplogroups observed in Tolima and Huila are listed in S2 Table. The absolute and relative haplogroup frequencies per department and for the whole region are reported in Table 1. The genotyping of 48 Y-SNP markers revealed six different major clades: R, Q, J, E, G, and T, representing 16 subhaplogroups (Table 1). More than 70% of the chromosomes in the total sample came from two clades: the European haplogroup R1b was found at the highest frequency (57.83%; n = 48), and the percentage of the Native American haplogroup Q1a2 was 16.86% (n = 14). No significant differences were found when the frequency distribution of haplotypes was compared between Tolima and Huila using AMOVA (*F*_ST_ = -0.00133; p = 0.50255+/-0.0016); these two groups were therefore were analyzed as one population.

**Table 1:** Y-chromosome haplogroup frequencies in the Tolima and Huila departments. The number of chromosomes per haplogroup is shown in parentheses (n).

Haplogroup-SNP	Huila	Tolima	Total
**E1b1b-M78**	4.76% (2)	2.43% (1)	3.61% (3)
**E1b1b-M81**	2.38% (1)	7.31% (3)	4.81% (4)
**E1b1b-M123**	2.38% (1)	0% (0)	1.20% (1)
**G-M201**	7.14% (3)	0% (0)	3.61% (3)
**J1—M267**	2.38% (1)	2.43% (1)	2.40% (2)
J2a-M410*(xM67, M92)	0% (0)	4.87% (2)	2.40% (2)
J2a-M67* (xM92)	2.38% (1)	0% (0)	1.20% (1)
Q1a2-M346*(xM3)	7.14% (3)	9.75% (4)	8.43% (7)
Q1a2-M3*(xM19, M194, M199)	9.52% (4)	7.31% (3)	8.43% (7)
**R1b-U106**	2.38% (1)	7.31% (3)	4.81% (4)
R1b- S116*(xU152,M529, M65, M153, M167)	28.57% (12)	34.14% (14)	31.32% (26)
**R1b-U152**	4.76% (2)	7.31% (3)	6.02% (5)
**R1b-M529**	14.28% (6)	7.31% (3)	10.84% (9)
**R1b-M153**	2.38% (1)	0% (0)	1.20% (1)
**R1b-M167**	2.38% (1)	4.87% (2)	3.61% (3)
**T-M70**	7.14% (3)	4.87% (2)	6.02% (5)
**Total**	100% (42)	100% (41)	100% (83)

The haplogroup diversity of the Tolima and Huila region was 0.8692. Haplotype diversity was evaluated using the information obtained from 17 STR loci, revealing a high diversity rate of 0.9680, characterized by 93% (n = 77) unique haplotypes. Shared haplotypes were observed for haplogroups E1b1b-M78, E1b1b-M81, Q1a2-M346*(xM3), R1b-U106, and R1b-S116*(xU152, M529, M65, M153, M167) (Table 2). The haplotype diversity per haplogroup was estimated for clades with at least five samples and was found to range from 0.904 for Q1a2-M346*(xM3) to 1.000 for R1b-U152, R1b-M529, T-M70, and Q1a2-M3*(xM19, M194, M199) lineages. This diversity analysis showed high genetic heterogeneity within haplogroups, with no signs of important founder events in the Tolima and Huila region.

**Table 2:** Diversity statistics per haplogroup in the Tolima and Huila region.

Haplogroup-SNP	Sample size (n)	N° Different Haplotypes	N° polymorphic loci	Average N° alleles per locus	Haplotype diversity^a^
**E1b1b-M78**	3	2	7	1.412±0.507	NA
**E1b1b-M81**	4	3	3	1.176±0.393	NA
**E1b1b-M123**	1	1	0	1.000±0.000	NA
**G-M201**	3	3	10	1.824±0.809	NA
**J1—M267**	2	2	10	1.588±0.507	NA
J2a-M410*(xM67, M92)	2	2	8	1.471±0.514	NA
J2a1-M67* (xM92)	1	1	0	1.000±0.000	NA
Q1a2-M346*(xM3)	7	5	6	1.412±0.618	0,904
Q1a2-M3* xM19, M194, M199)	7	7	16	2.941±0.966	1,000
**R1b-U106**	4	3	6	1.412±0.618	NA
R1b- S116*(xU152,M529, M65, M153, M167)	26	25	16	3.176±1380	0,997
**R1b-U152**	5	5	10	1.706±0.686	1,000
**R1b-M529**	9	9	12	1.941±0.827	1,000
**R1b-M153**	1	1	0	1.000±0.000	NA
**R1b-M167**	3	3	3	1.235±0.562	NA
**T-M70**	5	5	13	2.353±0.996	1,000
**Total**	83	77			

a. Haplogroup diversities were calculated for clades with five or more samples using Nei’s haplotype diversity [41].

NA: Not Applicable.

### Native American background of the Tolima and Huila populations

Most studies conducted in admixed Colombian populations have identified a male European contribution ranging from 58% to 94% and representation of Native lineages ranging from 1% to 38% [7,11,27,50].

The present study revealed a 16.86% frequency of Y-chromosomes belonging to haplogroup Q (Table 1). Individuals carrying the M3-derived allele, which characterizes the Native American founder haplogroup within Q1a2 [51], were found in seven samples, at a frequency of 8.43%, and all of these individuals exhibit unique haplotypes (Table 2).

Haplogroup Q1a2-M346*(xM3) was identified in the same proportion as haplogroup Q1a2-M3. This haplogroup has been found in samples from Argentina, Chile, Bolivia, some studies from North America, the northern part of South America, and Siberia [52,53].

**Fig 2:**
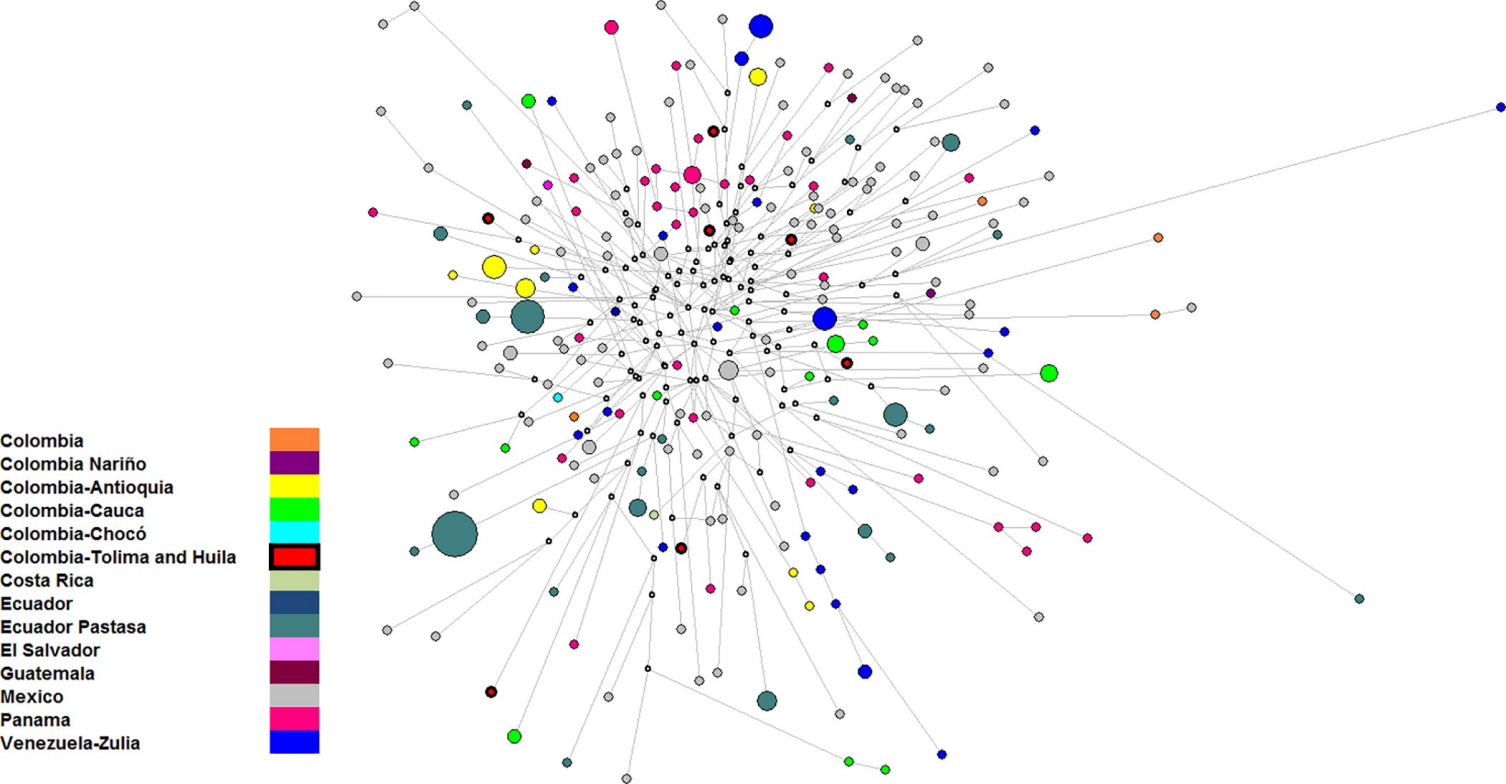
Median-joining network of haplotype Q1a2-M3 in 14 samples from Latin American population based on 15 STR markers. In the figure, the circles represent haplotypes, with areas proportional to their frequencies, and the colors indicate the original population. The median vectors (absent or extinct haplotypes) are shown in white.

Using a phylogenetic approach, the possibility of past minor founder effects was evaluated in the Native American lineages. In the Q1a2-M3*(xM19, M194, M199) network (Fig 2), 310 haplotypes belonging to six Colombian and eight Latin American populations were included (S1 and S3 Tables). Fig 2 shows that the seven sampled haplotypes are dispersed within the network, with no coincidence between them or with the other haplotypes analyzed. This finding reflects the high diversity among M3 Native American chromosomes at the haplotype level as well as the need to improve the analysis of new SNP mutations within this haplogroup to extensively characterize lineages and identify possible settlement routes of M3 carriers.

As illustrated in Table 2, the Q1a2-M346*(xM3) clade exhibited a diversity value of 0.904, with five different haplotypes, which are distinct from each other due to six polymorphic loci. A detailed analysis showed that all seven individuals carried allele 6 at the DYS391 locus. This allele is present in a very low frequency worldwide and can be used to extensively characterize the Q1a2-M346*(xM3) lineages. Among the 197,102 minimal haplotypes in the YHRD database (2018-03-06), only 334 show this allele (0.169%), which is mainly present in Asian and Latin American samples. In this study, the presence of this allele was confirmed via direct sequencing, as shown in S1 Fig.

**Fig 3:**
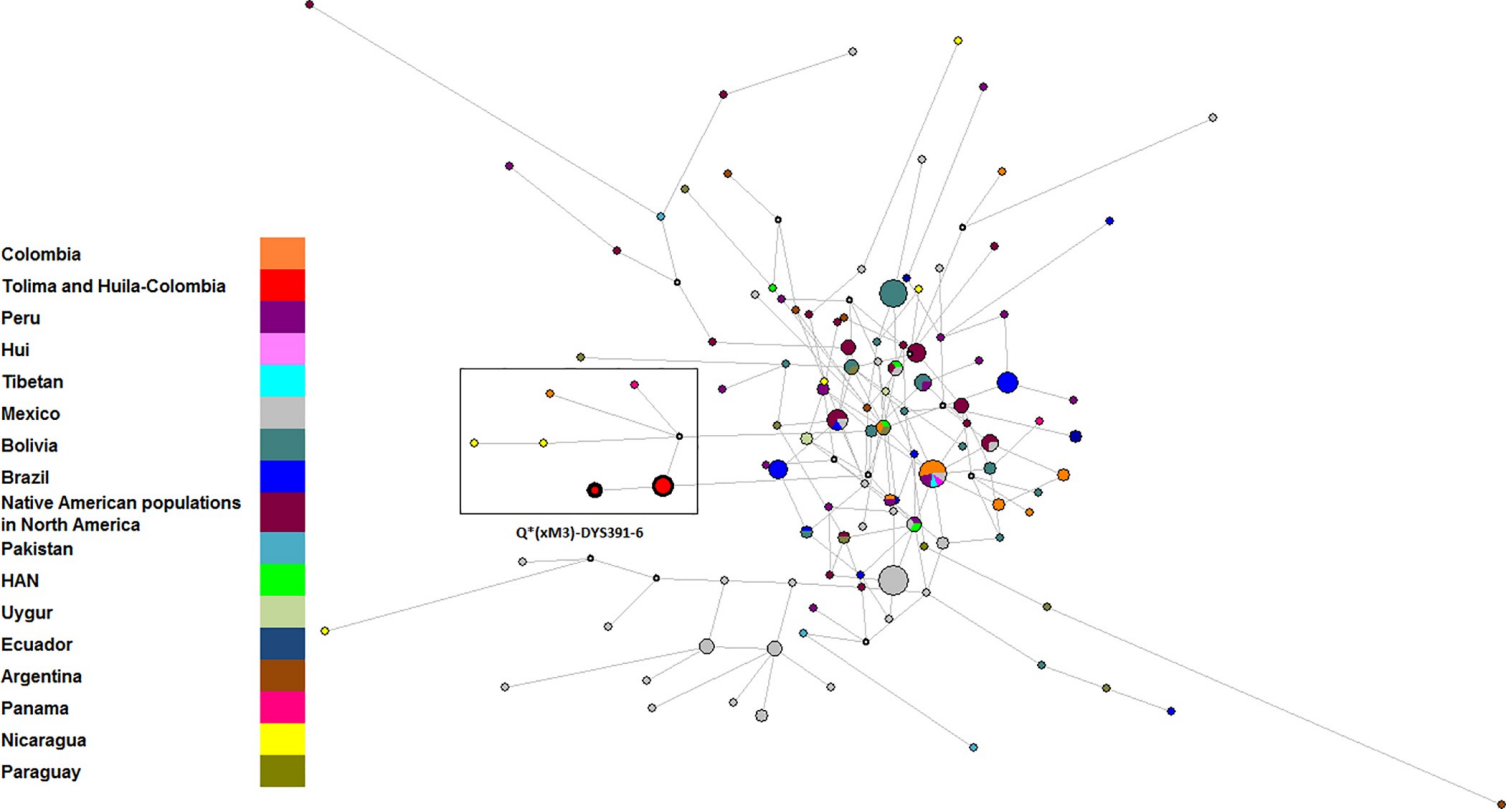
Median-joining network of haplogroup Q-M242*(xM3) using seven STRs (DYS19, DYS389 I and II-I, DYS390, DYS391, DYS392, and DYS393) to compare 17 Asian and American populations. The circles represent haplotypes, with areas proportional to their frequencies, while the colors indicate the population. The median vectors (absent or extinct haplotypes) are shown in white.

To identify patterns and matches between the unconventional Q1a2-M346*(xM3) DYS391*6 samples from the Tolima-Huila territory, a phylogenetic analysis was performed, using 193 previously reported Q-M242*(xM3) haplotypes from Asian and American populations (Fig 3 and S1 and S4 Tables). As shown in Fig 3, the seven Q1a2-M346*(xM3) haplotypes clustered with nine previously reported haplotypes (two from Nicaragua, one from Colombia, five from the Ngöbé-Native group of Panama, and one from non-Native individuals in Panama), all of which exhibit the allele DYS391*6 (S4 Table). This cluster forms a separate branch in the network, suggesting a closer evolutionary relationship among these Central and northernmost South American samples.

### New insights into the Peopling of Central America and the Northernmost Region of South America

The above analysis revealed high diversity in the Tolima and Huila Native American lineages. The Y-chromosomes from this study belonging to Q1a2-M3*(xM19, M194, M199) and Q1a2-M346*(xM3) did not match samples from East Asia or from the American continent, including Northern, Central, and Southern populations.

The results obtained from Q1a2-M346*(xM3)-DYS391*6 Y-chromosomes fit a model of isolation after colonization, which most likely erased the footprints of this lineage dispersion route from modern Native populations. This finding could indicate already extinct haplotypes or very low-frequency haplotypes in the current Native communities that have not been reported previously.

Nevertheless, DYS391*6 is not a private allele of a subhaplogroup within Q-M242*-(xM3); it has also been reported in individuals carrying the C-M130, O2-M95, Q-M242, Q-M3, and Q-M120 haplogroups, who mainly belong to the Han ethnic group, from Southeast Asian populations [54]. However, it is important to highlight that all DYS391*6 samples from Latin America with available Y-SNP data were classified within the Q-M242 clade [28,30,55,56,57,58, and this study].

For this reason, a broad search was conducted using the YHRD database (http://yhrd.org/) [46], and 334 haplotypes with this allele were found, including 300 from Asia (mostly in China), three from Europe, four from Australia, and one from the United States of America, while the remaining 24 were from Latin America. Among these 24 haplotypes, only six came from a Native population (Ngöbe in Panama), while two came from Nicaragua, one from Maracay-Venezuela, and the remaining 15 from admixed Colombian populations (Santander = 3, Bogota = 3, Valle del Cauca = 3, San Andres Island = 2, Antioquia = 1, Cundinamarca = 1, Nariño = 1, and Risaralda = 1).

Furthermore, related studies have obtained data from Central and South American samples. Battaglia et al. [57] reported three additional haplotypes, one from Colombia and two from Panama; Grugni et al. [58] added four samples from Panama; Jota et al. [30] detected the same allele in eight Coyaima individuals belonging to the “Pijao ethnic group” from the Tolima department; and Franco-Candela and Barreto [28] found an additional five haplotypes with this allele in another sample from the Coyaima Native group.

Nevertheless, many of the reported DYS391*6 samples were not assigned to a haplogroup; however, their haplotype information is valuable for assessing similarities and differences among these chromosomes. To evaluate the similarity among the DYS391*6 haplotypes found in the Asian and Latin American data, a phylogenetic network was created (S5 Table and S2 Fig). Based on this analysis, a different origin was identified for Asian and Latin American samples as well as a close connection between Central and South American DYS391*6 haplotypes.

In 2013, Battaglia and collaborators [57] suggested that all Native South American chromosomes classified as Q1a2-M346*(xM3)/Q-M242*(xM3) should be allocated to the Q1a2-L54*(xM3) haplogroup, representing the second largest Pan-American founder Y-chromosome lineage, including those carrying allele 6. Similarly, to identify new informative Y-SNPs of subclades within haplogroup Q1a2-L54* (xM3), Jota et al. [30] analyzed 1841 Native individuals from South America within this haplogroup, including eight Coyaima samples from Tolima. Based on these Coyaima data, two new Q1a2-L54*(xM3)-derived sublineages were identified, Q-SA03 (n = 7) and Q-SA02 (n = 1), both of which were found exclusively in this Native Colombian group.

The Coyaima Cariban linguistic family is one of the tribes belonging to the Pijao Native American group, which is only found in the Tolima department. When 15 Y-STRs were used to compare the haplotypes from our Q1a2-M346*(xM3)-DYS391*6 samples with the Coyaima data reported by Jota et al. [30] classified as Q-SA03 and Q-SA02, complete matches were found between four individuals. The same analysis was performed for the Coyaima sample from Franco-Candela and Barreto [28], in which all individuals were reported as Q-M242*(xM3). In this case, there was not a complete haplotype match. However, the samples showed differences in only one or two microsatellites. Therefore, a close genetic relationship between our data and the other two publications was confirmed (S6 Table).

**Fig 4:**
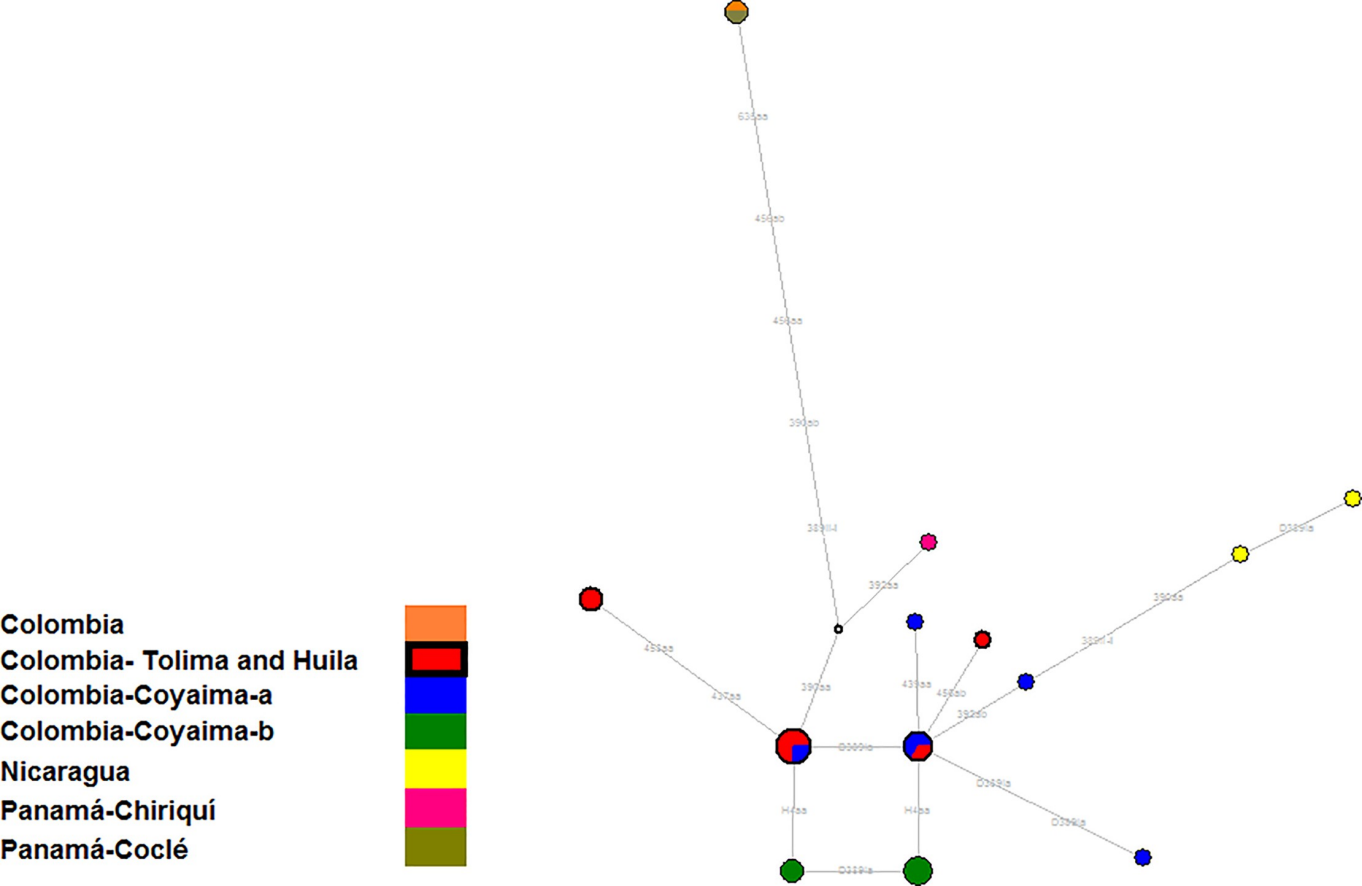
Median-joining network of 25 Q-M242*-(xM3)-DYS391*6 South American Y-chromosomes using 15 loci STR. The circles represent haplotypes, with areas proportional to their frequencies; the numbers of haplotypes in each node are given in the figure mutation positions are shown; and the colors indicate the population of origin. Coyaima-a* represents the data reported by Jota et al. [30]. Coyaima-b* represents the data reported by to Franco-Candela and Barreto [28]. The median vectors (absent or extinct haplotypes) are shown in white.

The phylogenetic relationships reported to date between all people in the Central and South American populations with allele six at DYS391 who were typified as Q-M242*(xM3) were further analyzed using YFiler profiles (S6 Table). In this network, 13 different nodes representing a total sample of 25 individuals were found; the results are shown Fig 4, and the genetic information is provided in S6 Table.

From this analysis, there were three main findings: (i) the Tolima and Huila region is characterized by the highest frequency of Q1a2-M346*(xM3)-DYS391*6 chromosomes (21 out of 25 haplotypes); (ii) 50% of these 25 haplotypes are different (N = 13); and (iii) the haplotype variability of these characteristic chromosomes is high in the Tolima and Huila samples, with nine of 13 Y-haplotypes differing.

These results indicate the middle Magdalena River region as the principal zone within all of Central and South America where this particular Native lineage is found. It is possible that all of the collected samples belong to the new sublineages Q1a2-SA02 and Q1a2-SA03 reported by Jota et al. [30]. However, to confirm this hypothesis, it would be necessary to genotype L54 and these two new Y-SNPs in all Central and South American samples carrying the DYS391*6 allele. The time to the most recent common ancestor (TMRCA) for these 25 Q-M242*(xM3)-DYS391*6 Latin chromosomes is estimated to be 1,809 years (+/- 0,5345 years).

Additionally, it is fundamental to bear in mind the frequency of this allele in Panama. As mentioned above, this population exhibits 12 reported haplotypes, six of which are found in the Ngöbe Native group (these data were not included in the comparison because less than 10 STRs were genotyped). Panama is the country with the second-highest frequency of the Q*-DYS391*6 lineage. Grugni et al. [58] proposed that the Isthmo-Colombian area is where this clade first arose.

The Isthmo-Colombian area has been described by O'Connor and Muysken [59] as a territory that was dominated by speakers of Chibchan languages for thousands of years. These Native individuals were distributed across four noncontinuous regions in Central and South America: eastern Honduras; from southern Nicaragua to western Panama; eastern Panama and northwest Colombia; and along the Magdalena River from Cundinamarca, Colombia northward to the Caribbean Sea. Therefore, this scenario is a plausible hypothesis in light of current evidence showing that all samples carrying DYS391*6 from the continental part of Colombia come from the Andean region.

In addition, a recent study of ancient human bones belonging to a Native Colombian group belonging to the Chibcha “Muisca” family from the IX to XVI centuries A.D. in the eastern Colombian Andes identified one male carrying allele 6 at locus DYS391 [60]. Based on this archeological finding, it is possible to confirm the presence of this allele in the genetic pool of Native Chibchan Nativepeople dating back to prehistoric times.

The narrow Isthmus of Panama is a likely bottleneck that should have allowed only a comparatively small number of nomadic hunters, fishermen, and gatherers to enter the northern Andean region via the Atrato, Cauca, and Magdalena rivers and tributaries through the northern reaches of the Colombian mountain ranges belonging to the greater Andean mountain range [59,61].

After subsequent demographic events such as isolation, genetic drift, and gene flow between Native American populations, speakers of different languages, such as Chibcha and Cariban languages, could have initiated the incorporation of this new Native lineage among local settlers of the middle Magdalena River region. Their descendants then survived through colonial times and waves of violence during the XIX and XX centuries. Today, they are still present at a high frequency in the Tolima and Huila departments and are part of the Colombian genetic pool.

### European lineages

The influx of male Europeans represented the greatest legacy in both the Tolima and Huila departments (83.14%). These haplotypes were distributed among 14 different subclades, within haplogroups R, E, G, J, and T (Tables 1 and 2).

Haplogroup R1b-M269 was the most frequent in our sample (57.83%; n = 48), which was characterized by high haplogroup diversity, including six sublineages: R1b-S116*(xU152, M529, M65, M153, M167), R1b-U152, R1b-M529, R1b-M153, R1b-U106, and R1b-M167 (Table 1). Additionally, the R1b sublineages showed a high diversity rate, with just one shared haplotype within the R1b-U106 and R1b-S116 haplogroups. The greatest numbers of polymorphic loci were found within subclades R1b-S116, R1b-U152, and R1b-M529, with 16, 10, and 12 polymorphic loci, respectively (Table 2).

These results regarding haplogroups and diversity reflect the migration dynamics during colonization and at the end of the XIX century in Tolima and Huila. These dynamics were characterized by the arrival of settlers from various parts of Europe, mainly from the Iberian Peninsula (i.e., Basque Country, Spain, Italy, and France). In that region, the presence, frequency, and diversity levels of these R1b-M269 sublineages are similar to those reported from this analysis [62,63,64,65,66].

Other frequent haplogroups common in western Europe, such as J members (n = 5, Table 1), were also identified in Tolima and Huila, which may be related to the above historical migratory movements. Haplogroup J has been subdivided into two major clades, J1 and J2. Both haplogroups were detected based on the M267, M410, and M67 mutations. This haplogroup evolved in the ancient Near East and was carried into North Africa, Europe, Central Asia, Pakistan, and India [67,68,69,70].

Eurasian clades T-M70 and G-M201 were also present, at frequencies of 6% (n = 5) and 3.61% (n = 3) respectively, neither of which showed shared haplotypes (Table 2). Haplogroup T-M70 is one of the most widely dispersed paternal lineages in the world. Haplogroup G-M201 is common in the Middle East, the Mediterranean, and the Caucasus Mountains [71].

### African ancestry

Haplogroup E, defined by the M96 mutation, is the most common human Y chromosome clade found in Africa [72]. Within this haplogroup, E1b1b shows the widest geographic distribution, being present in North Africa, West Asia and southern Europe [73,74,75]. Three haplogroups within the E1b1b clade were found in the Tolima and Huila territory: E1b1b-M78 (n = 3, 3.61%), E1b1b-M81 (n = 4, 4.81%), and E1b1b-M123 (n = 1, 1.20%) (Table 1). It is more likely that these haplogroups were introduced by Europeans, since they have been reported at relatively high frequencies in Iberian populations as a result of Muslim invasions [74].

The most common sub-Saharan African lineage, E1b1a-M2 [72], was not detected in the sample.

From the point of view of Colombian history, this is an unexpected result, because approximately 200 thousand slaves arrived in the Colombian territory through the port of Cartagena during the colonial era [76] and then dispersed via the Magdalena and Cauca Rivers to other countries or were sold in Colombian markets. One of these internal markets was Honda City in the Tolima department.

However, in the last population census of Tolima and Huila, only approximately 38 thousand people (1.8% of the total population in both places) were reported as "Afro-Colombians" [77].

Hence, the absence of this lineage in our sample might be an effect of sample size due to its low population frequency. This low representation may have resulted from the high proportion of European men present in the region, which might have reduced the number of descendants carrying haplogroup "E1b1a" in this area due to their frequency.

### Genetic relationships with other colombian populations

**Fig 5:**
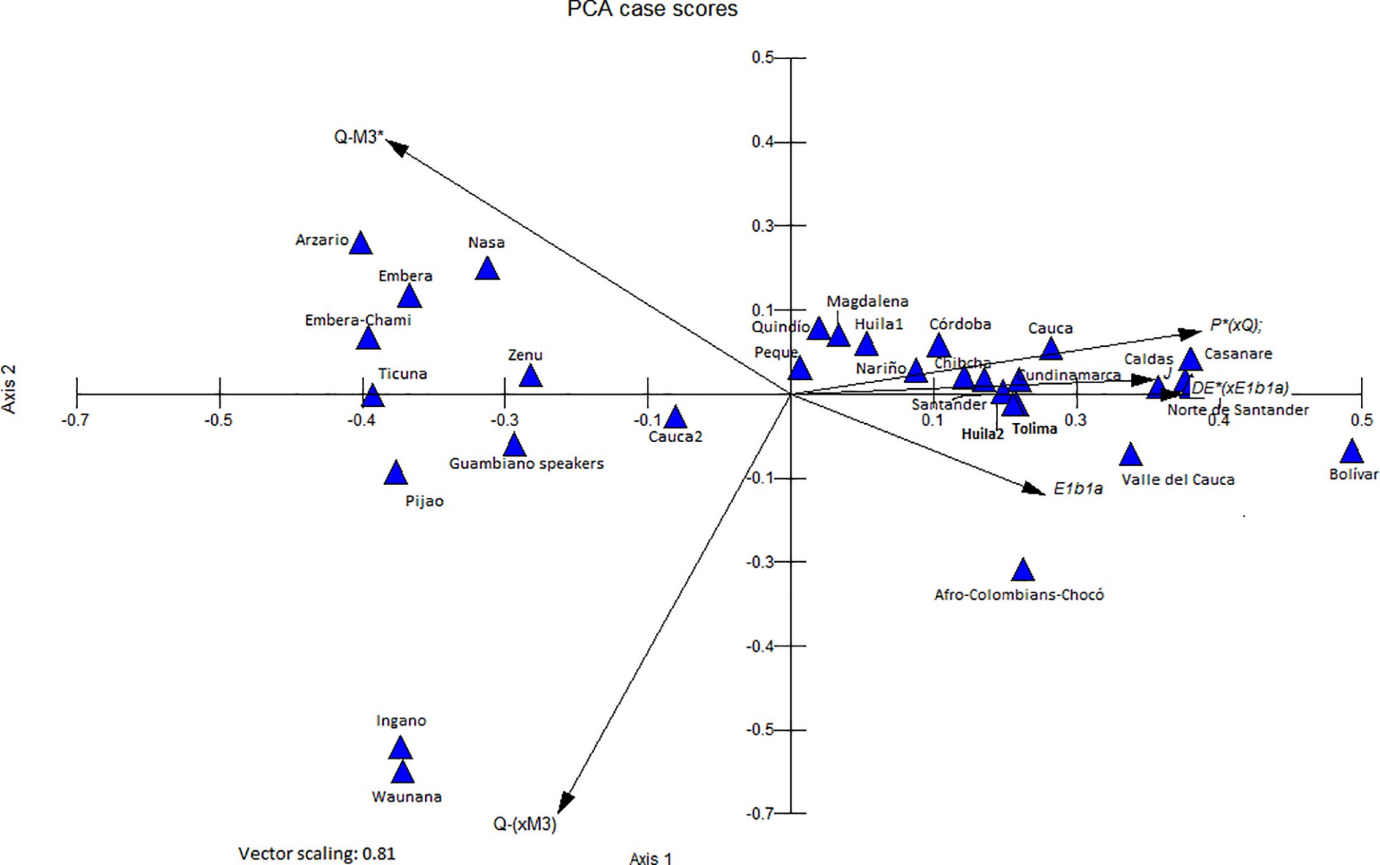
Principal component analysis for 32 Colombian populations using the haplogroup frequencies of Q-M242*(xM3), Q1a2-M3, P*-92R7(xM167), J-12f2a(xM9), DE*-YAP(xM2), and E1b1a-(M2). A total of 66.46% of the observed variance is explained by the first two axes represented in this figure.

Based on the data provided in S7 Table, PCA was performed to visualize which Colombian populations share frequencies and which haplogroups generate higher separations given their representations. These results are shown in Fig 5.

In the presented diagram, the non-Native populations cluster in the first quadrant of the plane, along with the Tolima and Huila populations (corresponding to the populations examined in this study); this group is mainly characterized by high frequencies of European male lineages, including P*-92R7(xM167) and J-12f2a(xM9). In the case of Native populations, these clades are divided into two groups. The first is located in the second quadrant of the plot, where the frequency of Q1a2a*-M3 is close to one (Arhuaco, Kogi, Arzario, Emberá, Zenú, and Ticuna). The second group is located in the third quadrant, where Q lineages other than Q-M242*-(xM3) predominate (Waunana and Ingano). These results show clustering among the Tolima and Huila samples and other admixed Colombian populations, mainly based on the high prevalence of this European lineage, illustrating that the European influence is stronger in urban, rather than rural areas.

## Conclusions

The location of the Tolima and Huila departments in the center of the country, characterized by the rainforest biome [78] and its connection with the rest of Colombia via the Magdalena River, has made the region one of the most important peopling areas in Colombia. This territory has received waves of migration of people from all over the world, from pre-Columbian times to the present.

Although this territory does currently not include any major Colombian city and is instead composed of many rural municipalities, the Y-chromosome gene pool found in this study is indicative of a population with high diversity and similar frequencies of paternal lineages that are very common in European regions such as Spain or the Iberian Peninsula. This diversity is a consequence of the economic and political historical importance of this region during colonial times and in the XIX century.

It is important to note the lack of Y-chromosomes belonging to haplogroup E1b1a-M2 in our sample. This finding does not preclude the possibility that there are descendants of this lineage in the Tolima and Huila departments. Men with this genetic inheritance do exist in the territory, albeit not in high frequencies. The probability of sampling these individuals is therefore lower than for other, more frequent lineages.

The analysis also revealed uncommon Native American haplogroups at high frequencies. All archeologic and genetic evidence suggests that the Q1a2-M346*(xM3)-DYS391*6 lineage arose in prehistoric times in the Isthmo-Colombian area. However, after many generational and demographic events, this lineage currently only appears at a high density in and could even be considered near-exclusive to the Tolima and Huila departments. This hypothesis can be confirmed after analyzing more territories along the Colombian Andes in depth.

For this reason, it is important to assess new informative SNPs within the Q1a2 clade in Native and non-Native communities to obtain new insights and clues about the origin and dispersion routes that Native American groups have followed throughout time.

## Supporting information

S1 Fig Direct sequencing of the DYS391 system, showing the allele 6. (PNG)

S2 Fig Median-joining network of haplotypes DYS391*6 found in Asia and Latin American samples. **The comparison was made using 15 STR markers.** Circles represent haplotypes, with areas proportional to their frequencies; colors indicate the population of origin. The median vectors (absent or extinct haplotypes) are shown in white.
(TIF)

S1 Table Population data sets used for Y-STR comparative analyses. (XLSX)

S2 Table List of Y-chromosome SNP haplogroups and STR haplotypes found in samples from the Tolima and Huila regions. (XLSX)

S3 Table List of the Y-STR haplotypes belonging to the haplogroup Q1a2-M3 among the Central and South American populations considered in the network analysis. (XLSX)

S4 Table List of the Y-STR haplotypes belonging to the haplogroup Q1a2-M242*(xM3) among the Central and South American populations considered in the network analysis. (XLSX)

S5 Table Y-chromosome haplotypes associated with the DYS391-6 allele among Asian and Latin American populations. (XLSX)

S6 Table Y-chromosome haplotypes associated with the DYS391-6 allele typified as Q-M242*(xM3) in Latin American populations. (XLSX)

S7 Table Absolute frequencies of Y-chromosome haplogroups and sub-haplogroups in the 30 Colombian samples included in the PCA. (XLSX)

S1 File Historic cartography of the Tolima and Huila departments. (DOCX)
