# Supplementary figures and images for: Paternal portrait of populations of the middle Magdalena River region (Tolima and Huila, Colombia): New insights on the peopling of Central America and northernmost South America
Source: PLoS One. 2018 Nov 15;13(11):e0207130. doi: 10.1371/journal.pone.0207130 (PMC6237345; doi:10.1371/journal.pone.0207130)

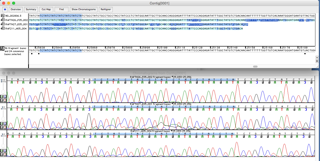

Supplement: S1 Fig — (PNG) [file pone.0207130.s001.png]

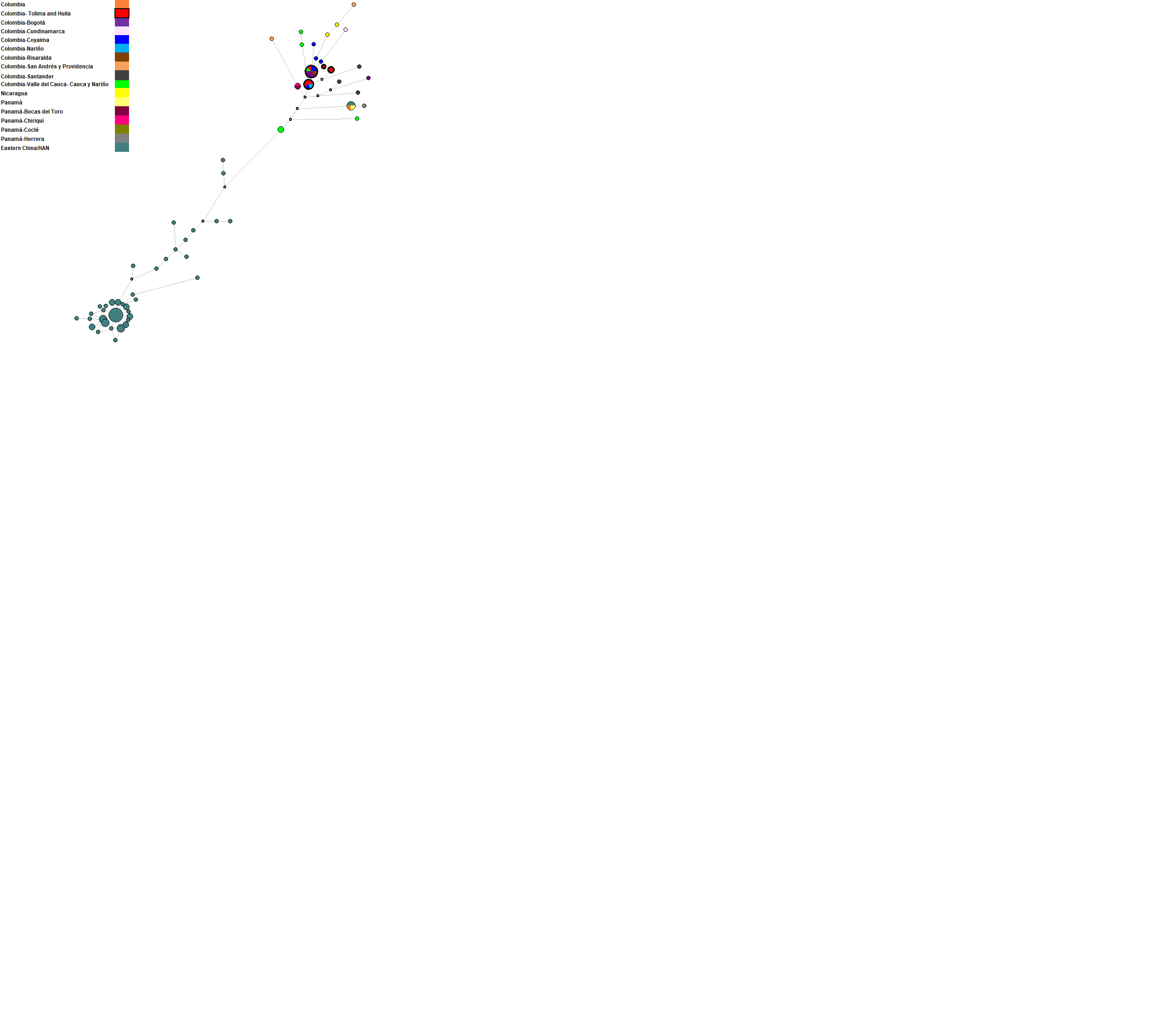

Supplement: S2 Fig — The comparison was made using 15 STR markers. Circles represent haplotypes, with areas proportional to their frequencies; colors indicate the population of origin. The median vectors (absent or extinct haplotypes) are shown in white. (TIF) [file pone.0207130.s002.tif]
