# Supplementary material for: Paternal portrait of populations of the middle Magdalena River region (Tolima and Huila, Colombia): New insights on the peopling of Central America and northernmost South America
Source: PLoS One. 2018 Nov 15;13(11):e0207130. doi: 10.1371/journal.pone.0207130 (PMC6237345; doi:10.1371/journal.pone.0207130)
Supplement: S1 File — (DOCX) [file pone.0207130.s010.docx]

As was mentioned in the main body of the article, prior to the field phase, a historic cartographic analysis was performed for both departments to identify the main geographic sites where the paternal lineages that have shaped the region have gathered over time. This historic cartography was constructed based on secondary sources of information and analysis of the available information on each one of the 47 and 38 municipalities that constitute the Tolima and Huila departments, respectively. This information has been recorded from pre-Columbian times to the present, in terms of focusing on the following variables:

a. Historic and Archeological records: in this item, we searched for information about pre-Hispanic settlements, Spanish colonization, the founding of settlements and cities, mass displacement of the population, natural disasters, the historical evolution of the cities, growing populations, and the most important surnames in each municipality.

b. Ethnographic information: in this, there was awe focused on the history and evolution of the current native communities in the zone was made. We followed by following their sites of settlement places and population migration dynamics.

c. Economics: we were interested in all information related to large landholders and large farms, or “latifundiums", and as well as any economic activity (agricultural, mining, commercial and services activities) which that could have led to the generation of new populations.

d. Geographic: we outlined the main routes used by people in each municipality to develop their activities and connect the departments with other Colombian populations. Furthermore, we analyzed the existence of geographic and political barriers among different populations.

e. Demographic: we took into account variables such as the density of the population, percentages of migration and emigration, and birth and death rates.

All of these data were registered in a database and later analyzed. A map was designed in order to visually subdivide the Tolima and Huila departments according to historical evidence of the ethnic origin of migrating groups and migration events. The following seven zones have been distinguished (See map No. 1):

Antioquia ancestrally zone: Composed of the municipalities in which the majority of the inhabitants have a recognized “Antioquia ancestry” dating back to the late nineteenth century. As is mentioned by Carvajal-Carmona et al. (2000), the Antioquia background refers to a regional identity of a Colombian group (termed paisa) that has developed around and within the province of Antioquia since the mid-17th century. Today, in addition to the Antioquia department, this sociocultural group is also found in the northwestern region of Tolima – the mountainous region. This area includes the eastern part of the Tolima, which has been divided into the Nevados Subregion. In terms of its genetic legacy, Rishishwar et al. (2015) reported that the primary ancestry component of this group is European (average = 74.6%, range = 45.0%–96.7%), followed by Native American (average = 18.1%, range = 2.1%–33.3%) and African (average = 7.3%, range = 0.2%–38.6%). In terms of its Y-chromosome contribution, Rojas et al, (2010) reported 94% of European haplogroups, 4% of African haplogroups and 2% of Native American lineages.

Corridor area: This area is composed of the municipalities located in the northeastern area of Tolima and the Magdalena River. This zone was once an important economic corridor for large amounts of both national and international commerce, due to its advantageous commercial position in the nineteenth century. This corridor has had experienced great population flows from different parts of Colombia, especially from the department Cundinamarca department, which includes Bogota, the capital city of Colombia. From the genetic Y-chromosome point of view, Hidalgo, (2015) reported for Cundinamarca that more than 90% of haplogroups in Cundinamarca that belong to European haplogroups and, while only 7% show of Native American ancestry.

Ancestral population area of Tolima: The two previously described areas had historical evidence of communication and population movements from other parts of the country to the Tolima department. This area, however, is based on the municipalities that were influenced by other regions but that have maintained their peasant population that since the colonial times, and the subsequent admixture process has occurred in these territories. Here, all the municipalities of central Tolima stand out, as well as do the mountainous regions that were colonized by Tolima’s original population at the beginning of the XX century in the southwestern and eastern areas. In this territory, Ibague, the capital city of the department, has been the only place that has been studied from the genetic perspective, from. In this population, Criollo, (2012) reported the following admixture frequencies based on a characterization of 100 AIMs the following admixture frequencies: 42.72% of Native American, 2.32% African and 54.96% European. From the According to male inheritance, the same author reported 11% for Native haplogroups, 87% of European clades and 11.1% of E haplogroups.

Pijao zone: This area is composed of the municipalities of both departments that include Pijao ethnic group reservations or cabildos of the Pijao ethnic group, either from colonial times or recently granted. In turn, there are some places in this area that have experienced large displacements of their indigenous populations, generating new Pijao settlements across the region, even without these two territorial figures. Criollo (2012) studied the autosome and Y chromosome ancestry on native reservations and in other nearby municipalities, reporting and reported for this group an autosomal genetic legacy for the Y-chromosome in this group of 69% of Native ancestry, 26% of European lineages and 3.7% of E lineages. For the autosomal landscape, he found the following proportions: 84.8% of Native American, 12.8% of European 2.4% of African contributions.

Ancestral population area of ​​Huila: The reasoning behind the creation of this zone is similar to that or the third zone, but of course, within the Huila department of course. The populations of the north and center of the department stand out; as well do the mountainous zones colonized in the XX century by farmers dedicated mainly to mainly cattle ranching and rubber and Peruvian bark extraction. This zone includes the northern and central regions of Huila, and the three municipalities of Pitalito, Palestina, and Acevedo, seeing as they exhibit the same colonization characteristics. In this zone, Neiva (the capital city of the Huila department) is the only one area that has been studied from the Y-chromosome genetic perspective, with Rojas et al, (2010) reported a 14.3% of Native American ancestry and 85.7% of European haplogroups.

Nasa zone: Composed by This area is composed of the municipalities of Huila that have included the Nasa indigenous population (also known as the Paez indigenous group), from colonial times up to recent times – especially since the Paez River avalanche in 1994, have included the Nasa indigenous population (known also as a Paez indigenous group). A good number of reservations and cabildos are located in this zone; they, and the associated individuals exhibit constant movements from their locations in the Huila, to the other Nasa communities in the Cauca department. In terms of genetic studies, Criollo, (2012) reported from the following findings in relation to Autosomal admixture: 95.7% Native American, 3.1% of European and 1.2% of African. For the Y-chromosome, the same author found 72.2% of Q-M3 members, 22.2% of European clades R1 and I, 2.8% of haplogroup E and 2.8% of other haplogroups.

Cauca and Nariño ancestry area: This area is composed of municipalities in the southwest of Huila that exhibit records of migrations and economic relationships from the Cauca and Nariño departments with this region. This zone is different because it is characterized by the population dynamics between the southwestern part of the department and other departments in the southern part of the country. There is not any available genetic research for this region.

This a priori classification of the population was established as a starting point to distinguish a series of differences within the region, which were verified at the time of going into the field.


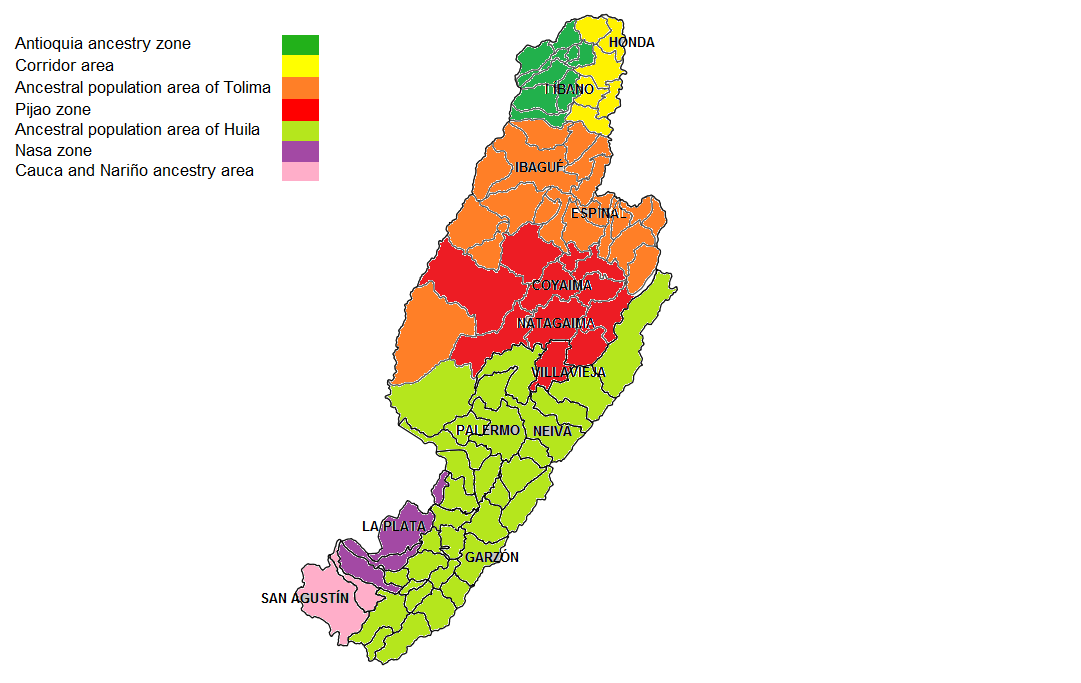


**Map 1.** Historic cartography of the migration groups that have shaped the gene pool of the Tolima and Huila regions over time. Map created with the QGis open source software, map data is available from https://geoportal.dane.gov.co/v2/?page=elementoDescargaMGN

**Bibliography**

Chala Aldana D. Lo histórico, lo genético y lo sociocultural, 3 perspectivas para diferenciar poblaciones: el caso de los departamentos del Tolima y el Huila (Colombia). (2012, Not published) pp.13-15.

Criollo A. Caracterización molecular de la variación genética en cuatro etnias indígenas (Pijao, Paez, Embera y Zenu) y dos poblaciones mestizas de colombia (Tolima y Córdoba) mediante marcadores del mDNA, NRY Y AIMs. M.Sc, Universidad del Tolima. 2012. Available from: <http://repository.ut.edu.co/handle/001/1087>

Hidalgo Cerón VF. Análisis de marcadores moleculares Y-SNPs para una población bogotana y su aplicación en procesos de identificación humana . M.Sc Thesis, Universidad Nacional de Colombia. 2015. Available from: <http://www.bdigital.unal.edu.co/49833/>

Rishishwar L, Conley A, Wigington C, Wang L, Valderrama-Aguirre A, King Jordan I. Ancestry, admixture and fitness in Colombian genomes. Scientific Reports [Internet]. 2015. doi: 10.1038/srep12376.

Carvajal-Carmona LG, Soto ID, Pineda N, Ortíz-Barrientos D, Duque C, Ospina-Duque J, et al. Strong Amerind/white sex bias and a possible Sephardic contribution among the founders of a population in Northwest Colombia. Am J Hum Genet. 2000;67(5):1287–95. doi: 10.1016/S0002-9297(07)62956-5

Rojas W, Parra MV, Campo O, Caro MA, Lopera JG, Arias W, et al. Genetic make up and structure of Colombian populations by means of uniparental and biparental DNA markers. Am J Phys Anthropol. 2010;143(1):13–20. doi: 10.1002/ajpa.21270

"QGIS Development Team (2018). QGIS Geographic Information System. Open Source Geospatial Foundation Project. [http://qgis.osgeo.org](http://qgis.osgeo.org/)".

Geoportal [Internet]. Descarga del Marco Geoestadístico Nacional (MGN). Departamento Administrativo Nacional de Estadística DANE; 2017 [cited 2018Aug21]. Available from: <https://geoportal.dane.gov.co/v2/?page=elementoDescargaMGN>
